# Pancytopenia: An Etiological Profile

**DOI:** 10.5505/tjh.2012.98360

**Published:** 2012-03-05

**Authors:** Vandana Raphael, Yookarin Khonglah, Biswajit Dey, Priyanka Gogoi, Ashim Bhuyan

**Affiliations:** 1 North Eastern Indira Gandhi Regional Institute of Health and Medical Sciences, Shillong, Meghalaya, India

## TO THE EDITOR

Pancytopenia is the deficiency of all 3 cellular elements of blood, resulting in anemia, leucopenia, and thrombocytopenia. Pancytopenia may arise due to a number of disease processes that vary according to population, age, nutritional status, and the prevalence of infections [1]. Pancytopenia is a common hematological condition of varied etiology; however, only a few studies on pancytopenia from the northeastern region of India have been published [2].

We analyzed the clinical details, hematological profile, and bone marrow findings in 80 patients that fulfilled the diagnostic criteria for pancytopenia between January 2007 and December 2009 in order to discern its etiology. The results of ancillary tests performed to confirm the etiology were also recorded The frequency of symptoms at presentation varied among the 80 patients, as follows: pallor (100%), fever (42.5%), splenomegaly (27.5%), hepatomegaly (25%), and bleeding (23.7%). Patients that were receiving chemotherapy for cancer were excluded from the study. Mean age of the patients was 30 years (range: 1-79 years) and the male:female ratio was 1:1.2. Megaloblastic anemia in adults and acute leukemia with aplastic/hypoplastic anemia in children were the most common causes (Table 1), as previously reported [3,4].

Pancytopenia is a common hematological condition encountered in clinical practice and has an extensive differential diagnosis. It should be suspected on clinical grounds in patients that present with unexplained anemia, fever, and a tendency to bleed. Bone marrow aspiration supported by biochemical investigation of such nutritional factors as serum iron, ferritin, vitamin B12, and folate levels, antinuclear antibodies, and serological tests for HIV and enteric fever will facilitate a correct diagnosis. Although it is not necessary to perform trephine biopsy in every case, it is of paramount importance in cases of aplastic anemia, which results in a dry tap. As such, routine bone marrow aspiration in every suspected case of pancytopenia is essential for diagnosis.

Several studies conducted on mainland India on the etiology of pancytopenia in adults and children have been published [1,3,5]. The present study was conducted in Meghalaya, a region of India in which the tribal population constitutes 85% of the total population and a traditional daily diet of rice, meat, fermented food, and green leafy vegetables is still followed, unlike in other regions of India. Dairy products and pulses are not part of the daily diet in the study region. Alcohol, fermented betel nuts, and tobacco are consumed by both men and women, irrespective of rural or urban habitation, level of income, and level of education [6]. In addition, malaria is endemic in the region. As such, the present study highlights the varied etiological pattern of pancytopenia in a region of northeastern India.

## CONFLICT OF INTEREST STATEMENT

The authors of this paper have no conflicts of interest, including specific financial interests, relationships, and/ or affiliations relevant to the subject matter or materials included.

## Figures and Tables

**Table 1:**
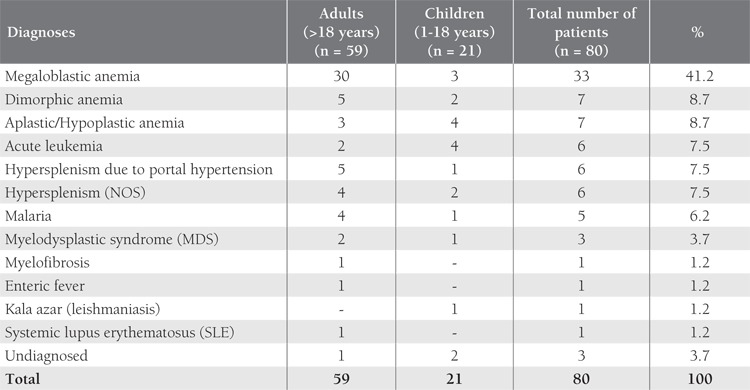
Etiological Profile of Pancytopenia
